# Box Experiment Study of Thermally Enhanced SVE for Benzene

**DOI:** 10.3390/ijerph18084062

**Published:** 2021-04-12

**Authors:** Qixiang Zhang, Qiyan Feng, Xueqiang Zhu, Mei Zhang, Yanjun Wang, Liu Yang

**Affiliations:** 1 School of Environmental Science and Spatial Informatics, China University of Mining and Technology, Xuzhou 221116, China; zqxcumt@126.com (Q.Z.); zhuxq0615@163.com (X.Z.); 2 Jiangsu Design Institute for Mineral Resources, Xuzhou 221000, China; zhangmei@cumt.edu.cn (M.Z.); wyj5011@126.com (Y.W.); yangliu672134@163.com (L.Y.)

**Keywords:** soil vapor extraction, benzene, thermal enhancement, soil gas

## Abstract

In order to describe the changes of soil temperature field, air flow field and remediation situation with time during the process of thermally enhanced SVE (soil vapor extraction), a remediation experiment of benzene contaminated soil with single extraction pipe was carried out in a box device. The results showed that the whole temperature of the system was raised to 80 °C in 4 h. 43% of benzene were removed in the first 2% of the extraction time. After 24 h, the repair efficiency was close to 100%. The device can efficiently remove benzene from soil. By continuously monitoring the parameters in the operation process of the system, the spatial distribution of temperature and soil gas pollutant concentration with time was plotted. It showed the benzene concentration distribution in the soil gas was more consistent with the temperature distribution before the start of ventilation, and the concentration of benzene in the soil gas dropped rapidly after ventilation, while the temperature distribution was almost unaffected. In the treatment of soil with a benzene content of 17.8 mg∙kg^−1^, when the soil gas benzene concentration is the highest at 180 min, the peak value is 11,200 mg∙m^−3^, and the average concentration is 7629.4 mg∙m^−3^.

## 1. Introduction

With the development of modernization, the central urban areas of many cities in China are undergoing the transformation of emigrating heavy industries and heavy polluting industries. In the process of transformation, the pollution of sites left by coal, machinery, chemicals, building materials, textiles, metallurgy, and other industries is more serious. The pollutants include heavy metals, petroleum hydrocarbons, PAH (Polycyclic Aromatic Hydrocarbons), PCB (Polychlorinated biphenyls), and various organic solvents. Additionally, some sites experienced compound pollution with multiple pollutants [1]. Contaminated soil not only reduces the survival rate of soil insects and bacteria, but also inhibits the growth of plants [2,3,4,5]. Organic compounds in the seepage zone may diffuse below the groundwater level [6], and the risk of steam intrusion in residential areas begins to appear [7,8], which pose a threat to water quality and human health and have received extensive attention for many years. Developed countries around the world have invested effort and resources in the remediation of soil and groundwater pollution continuously, to support and encourage environmental scientists to study the remediation of soil and groundwater. With the joint efforts of scientists from all over the world, various remediation technologies for soil pollution have emerged constantly. Among them, the soil vapor extraction technology (SVE) has high efficiency, reliability and economy for the removal of volatile organic pollutants in soil. It was highly recognized by the US Environmental Protection Agency and is regarded as a “revolutionary technology” and vigorously advocated [9]. It has been widely used in remediation of volatile organic pollutants in soil around the world.

SVE technology is affected by many factors such as soil air permeability, soil water content, aeration rate, and saturated vapor pressure. James et al. [10] established a local equilibrium linear isotherm and studied the relationship between soil air permeability and SVE purification time. The results showed that the soil air permeability had a huge impact on the removal. Soil moisture content had different effects on different types of soil. On the one hand, the increase of soil moisture reduced soil air permeability, which was not conducive to the volatilization of organic matter [11]. On the other hand, the increase in soil moisture reduced the degree of adsorption of soil particles to organic molecules and speeded up the process of pollutant treatment. Wilson and others [12,13] indicated that there was an optimal value for the gas flow rate, and there was no significant effect on the removal rate if the value exceeds this value. The gas flow rate calculated by the experiment greatly reduced the exhaust gas processing volume and reduce the treatment cost. Most importantly, the treatment efficiency of SVE is also significantly affected by the saturated vapor pressure of pollutants, and the key factor affecting saturated vapor pressure of organic pollutants is temperature. When the temperature is between 50 and 150 °C, SVE technology can improve the removal rate of pollutants and remove more types of pollutants. When the saturated vapor pressure of organic matter is more than 70 Pa, all pollutants can be removed by SVE [14]. In the last two decades, thermally enhanced soil vapor extraction (T-SVE) has been proved to be an effective technology for removing volatile organic pollutants from contaminated sites [15,16,17,18,19].

In the preliminary work, our team investigated the characteristic pollutants in eight remaining contaminated sites in Xuzhou, Jiangsu Province. According to the investigation, the soil pollutants exceeding the soil standard mainly include benzene, HCN, arsenic, cadmium, lead, benzo (a) anthracene, benzo (b) fluoranthene, benzo (a) pyrene, Dibenzo anthracene, and petroleum hydrocarbon C10-C36. Most of these pollutants exist in plots with a depth of 0–6.5 m in the soil. Among them, pollutants in some sites even exceed environmental protection standards by thousands of times. 

The existing T-SVE experimental studies are mainly one-dimensional and two-dimensional [20,21,22]. Three-dimensional experiments have attracted more attention in recent years. Tasca [23] combined ultrasound irradiation with the electrochemical generation of ∙OH by Nb/BDD anode to remove Chlorpyrifos from aqueous solutions in three- dimensional electrochemical reactor. Li Xiaoya [24] took the hydrocarbon-contaminated soil as the treatment object and carried out the research on the enhanced SVE technology based on resistance heating in the three-dimensional box device. The research showed that the space setting of the heating rod significantly affected the removal effect of pollutants. In this study, in order to describe the process of T-SVE in three dimensions, a larger box-type device and supporting system were designed and fabricated. The remediation of benzene contaminated soil was carried out by heat transfer enhancement method. The changes of temperature field, humidity air flow and tail gas pollutant concentration during extraction, and the spatial distribution of soil residual pollutants after remediation were analyzed three-dimensionally. 

## 2. Materials and Methods

### 2.1. Soil Preparation and Characterization

The soil to be treated was collected from residential areas around a typical industrial wasteland in Xuzhou. After picking out small stones and plant debris, the air-dried soil samples were ground. Then the ground soil was cleaned with deionized water to remove surface impurities, dried at 80 °C for 8 h, and then the soil sample was passed through a 30-mesh sieve. The soil property was analyzed by Jiangsu Design Institute for Mineral Resources. Silicon content test adopted the quality method, referring to the Chinese standard LY T 1253-1999. Its basic properties were shown in Table 1.

According to previous experience, the benzene dissipated during soil mixing was about 70%. To prepare 20 mg∙kg^−1^ contaminated soil, 20,000 mg of benzene was added to 450 kg soil.

Firstly, benzene was completely dissolved with 20,000 mL deionized water. Then, after a series of mixing and shaking with soil samples, the benzene was uniformly distributed in the soil. Then the soil was put into the device immediately. Randomly three copies of the prepared soils were taken, put into a sealed black glass jar, and immediately detected with gas chromatography. The benzene concentrations measured in three samples were 17.1, 17.7, 18.5 mg∙kg^−1^, with an average value of 17.8 mg∙kg^−1^.

### 2.2. T-SVE Device

**Figure 1:**
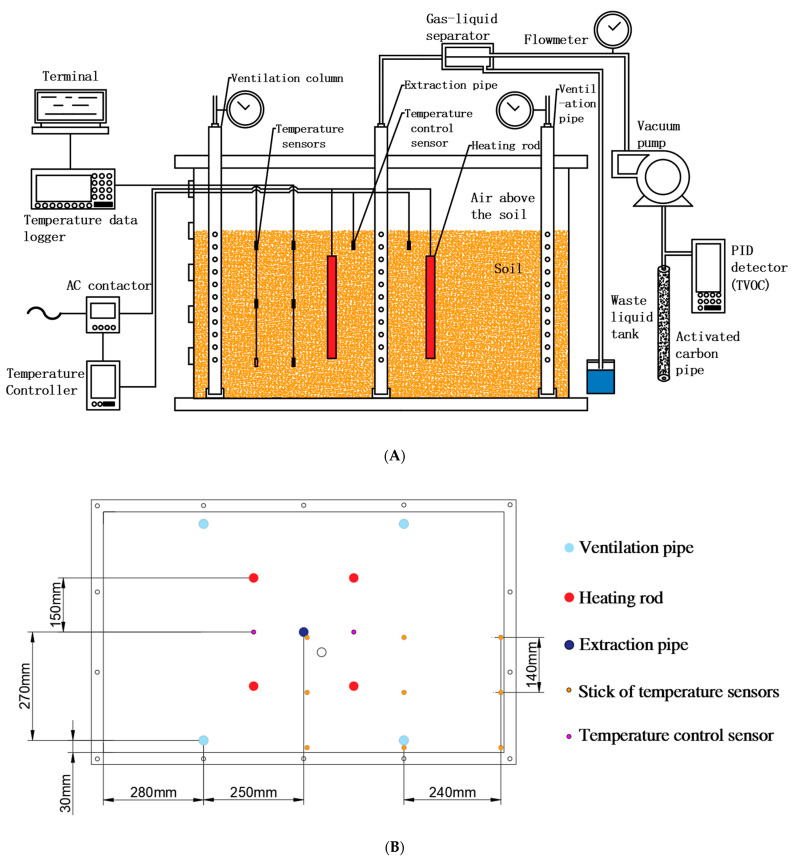
Schematic diagram of each unit setting (**A**). Plane distribution of each component (**B**).

According to the principle of soil vapor extraction technology, a thermally enhanced SVE box device was developed. A schematic diagram of each unit setting was shown in Figure 1A, and the plane distribution of related components during treatment was shown in Figure 1B. The simulation device consists of the following units: (1) the main structure: it is a stainless steel box with an internal volume of 1 × 0.6 × 0.6 m^3^, which has a thermal insulation layer, and is sealed except the opening of the extraction unit; (2) extraction unit: it includes a vacuum pump, extraction pipe, ventilation pipe, and matching pneumatic device. The extraction pipe has the same structure as the ventilation pipe, it is composed of an outer pipe for supporting and isolating soil and an inner pipe with good ventilation effect; (3) thermally enhanced unit: it is mainly the soil heating system. In this treatment, heating is carried out by thermal resistance. Relevant wires and sensor wires are connected through the glen head interface at the front of the device to ensure the airtightness of the device; (4) gas–liquid separation/treatment unit: mainly including gas collection pipeline, gas–liquid separator, waste liquid collection barrel, activated carbon pipe, etc. After the exhaust gas passes through the gas–liquid separator, the gas is discharged after being adsorbed by activated carbon, and the liquid is collected for centralized treatment; (5) control unit: it includes temperature controller and AC contactor, and uses a temperature control device to realize the automatic control of the heating rod. When the temperature of the temperature control sensor exceeds the target temperature, the heating rod is automatically turned off, and once the temperature drops below the target temperature, the heating rod is automatically turned on; (6) monitoring unit: including temperature sensor, data terminal of temperature sensor, glass fiber rod, electronic flowmeter, and benzene gas detector.

### 2.3. Process and Monitoring Method

The experiment adopted the mode of single extraction pipe, four heating rods and four ventilation pipes. The test ran for 24 h. As extraction significantly affects the temperature rise of the system, the treatment in the first 4 h was heated without ventilation, and the ventilation and extraction was started in the next 20 h. In the experiment, a single extraction pipe was used for air extraction, and four ventilation pipes were opened. The ventilation pipes were opened at the beginning of extraction.

The pneumatic pipeline connecting the extraction pipe was connected to the steam-water separator first, and then to the vacuum pump (FUJIWARA, Tokyo, Japan, 750 W 120 L∙min^−1^). The vacuum pump was set to the maximum power, and the flow rate reached 120 L∙min^−1^ when idling. The heating device adopted custom-made lotus root joint heating rod with 1000 W power, 3 cm diameter, 25 cm length, and 5 cm buried depth. The temperature control device adopted SIEVAL TC-05B (Guangzhou, China), it was connected with lotus root joint heating rod through AC contactor. The temperature control device was set to close the circuit breaker at 80 °C and opened below 70 °C. The embedded depth of the temperature control sensor was 5 cm.

The inflow rate and outflow rate data were acquired and stored by using the time-lapse shooting function of the camera. It was set to shoot once every 10 min to record the flow change every 10 min to represent the flow in 10 min. In addition, when monitoring the inflow rate, the inflow rate of four gas wells was recorded, respectively, to monitor the difference of ventilation in space.

After the experiment, the test soil in the device was taken out, and the concentration of residual pollutants was measured, so as to describe the distribution of pollutants. When sampling, it was divided into three layers, and sampling was carried out by using a long ring knife. A total of 25 sampling points were set, which were uniformly distributed in a rectangular shape on the plane. The collected soil samples were evenly divided into three layers on the depth, each layer was about 13 cm long. The sample was stored in 150 mL black plastic bottles. Sampling numbers: 1–25 from left to right on the plane, and Roman numerals I, II and III from top to bottom on the depth. A total of 75 soil samples were taken and the concentration of residual benzene was tested by gas chromatography.

Because the box cannot be opened during the experiment, the measurement of soil moisture was only carried out before and after the experiment. The soil moisture rapid tester was used to measure according to the soil sampling position after the test, and the measuring depths were 5, 20, and 35 cm.

The system temperature field was monitored by Coupling SH-X (Lianyi, Dongguan, China) system. A total of 27 sensors were used and divided into nine groups. Each group of sensors was tied to the glass fiber rod in three layers, so that the distance between the three sensors in each group and the bottom of the system in the longitudinal direction was 5, 20, 35 cm, respectively. The temperature field monitoring system continuously monitored the temperature field changes of the system to study the coupling between temperature field and pollutant concentration. The gas discharged from the vacuum pump was connected to an electronic flowmeter (SIARGO MF5706, Santa Clara, CA, USA) to monitor the real-time flow data, and then connected with a PID detector (mic-600, Kunliankeji, Guangzhou, China) with an accuracy of 1 ppm in parallel to monitor the benzene concentration of the exhaust gas in real time, and the detector was set to record the data once every 6 s. Most of the tail gas at the other end of the parallel connection was adsorbed by the activated carbon pipe.

The flow rate was recorded every 10 min to represent the average flow rate within 10 min, and the benzene concentration was recorded by the PID detector. Therefore, both the tail gas flow rate and the tail gas benzene concentration per second in the test can be expressed. The total amount of benzene extracted in each period can be obtained. The calculation formula is shown in Formula 1

(1) $$E_{i,n}=\sum_{i}^{n}C_{n}Q_{n}$$

$C_{n}$ is the benzene concentration in tail gas at time n;

$Q_{n}$ is the tail gas flow at time n.

In addition, to measure the variation of benzene concentration in soil gas, a gas sampler was used to sample the gas through 15 sampling ports at the front of the device. The tail end of the sampler was directly connected with a PID detector for pump sampling and real-time detection. It was read and recorded when the gas sampler enters the sampling port hole 10, 150, 300, 450, 590 cm. A total of 75 locations of soil gas benzene content were read in one period. Sampling times were 0, 20, 40, 60, 90, 180, 240, 250, 260, and 400 min after heating.

## 3. Results and Discussion

### 3.1. Distribution Changes of Temperature, Humidity and Soil Gas Benzene Concentration

**Figure 2:**
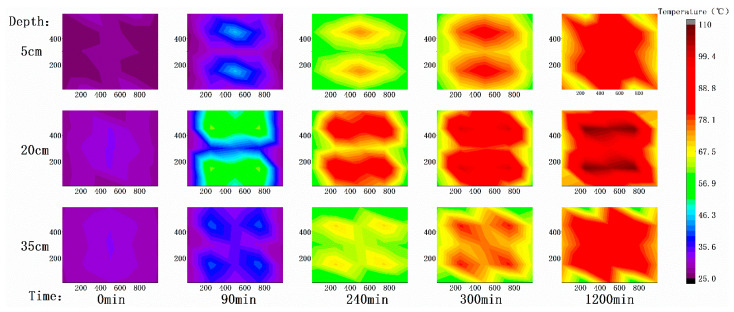
Isotherm map at 0, 90, 240, 300, 1200 min.

In order to simulate and monitor the specific change of temperature in space during the treatment process, 27 thermocouples were used to continuously monitor the temperature of the soil at depths of 5, 20, and 35 cm in the lower right part of the system. Data was recorded by the data terminal every 30 s. After screening, five typical time points (0, 90, 240, 300, 1200 min) were intercepted. Taking the left front end of the box as the origin, the monitored temperature in the lower right area was processed with central symmetry, and the isotherm diagram of each time point on the corresponding plane in the whole box was drawn to describe the change of temperature field during the experiment. The result is shown in Figure 2.

**Figure 3:**
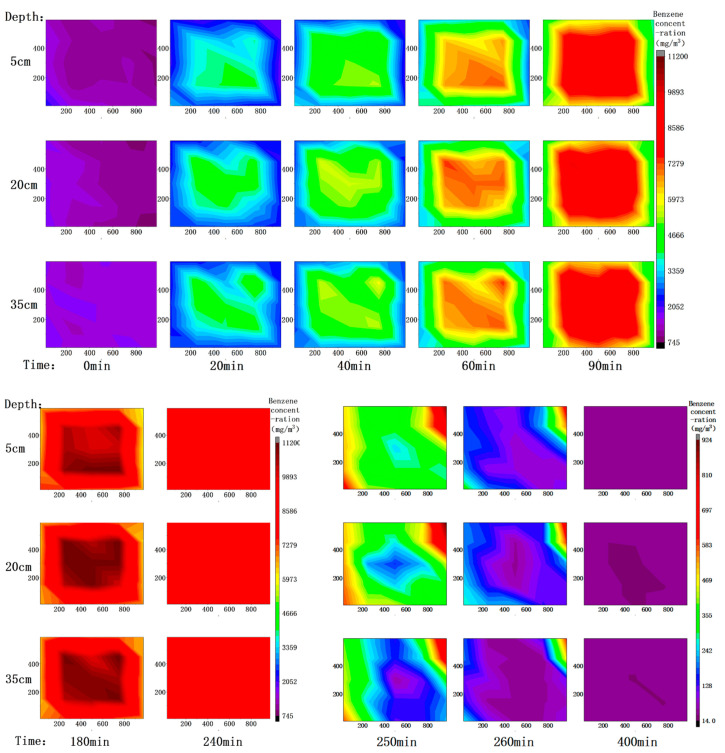
Contour map of soil gas benzene concentration at 0, 20, 40, 60, 90, 180, 240, 250, 260, and 400 min.

To describe the distribution of benzene content in soil gas over time, according to the results of soil gas measurement, the contour map of benzene concentration in soil gas was drawn in three layers (depth 5, 20, 35 cm) with the left front end of the box as the coordinate origin. The result is shown in Figure 3.

**Figure 4:**
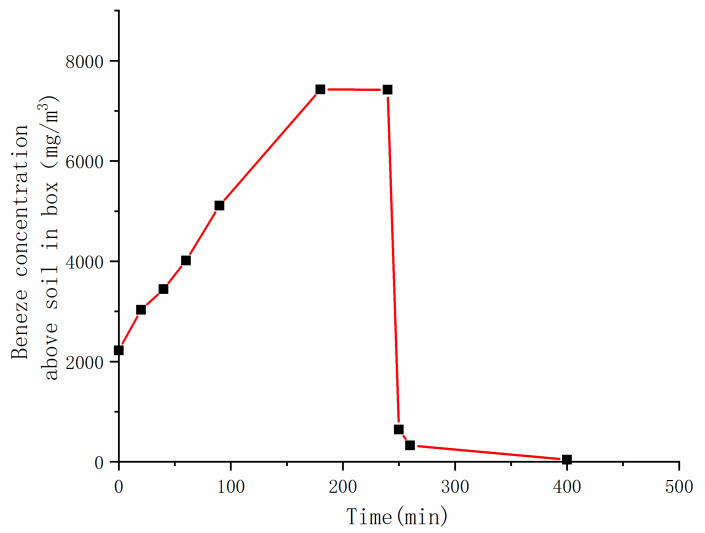
The benzene concentration above the soil in the box over time.

In addition, the benzene concentration in the air above the device was measured at the same time, as shown in Figure 4. The concentration above the device was basically proportional to time in the heating stage, and remained unchanged after reaching about 7500 mg∙m^−3^, it decreased rapidly after extraction. This was consistent with the benzene concentration in the exhaust gas. However, it was worth noting that the benzene concentration in the gas above the device was slightly lower than that in the exhaust gas.

**Figure 5:**
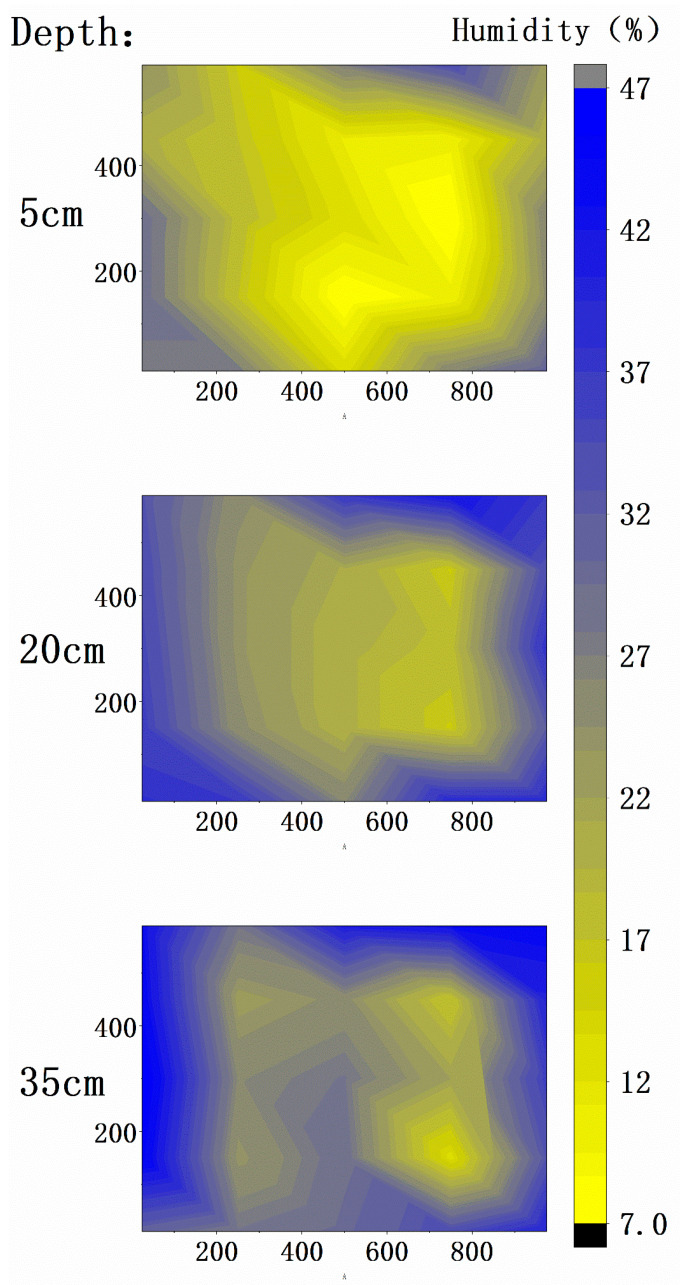
Distribution of treated soil moisture.

Before treatment, after repeated mixing, the soil moisture was almost uniform, which was 36%. After 24 h heating and 20 h extraction, the soil was divided into three layers (depth 5, 20, 35 cm), and the humidity was detected according to the soil sampling position. After treatment, the average humidity of the soil at the depths of 5, 20 and 35 cm was 20.28%, 29.84%, 32.56%, respectively, the overall average humidity was 29.39%. The treatment process took away 6.61% of the soil moisture, which was 14.54 kg. The contour map of humidity was drawn with the left front end of the device as the origin, and the result was shown in Figure 5. The soil moisture after treatment was low in the surface layer, high in the bottom layer, low in the middle, and high around.

**Figure 6:**
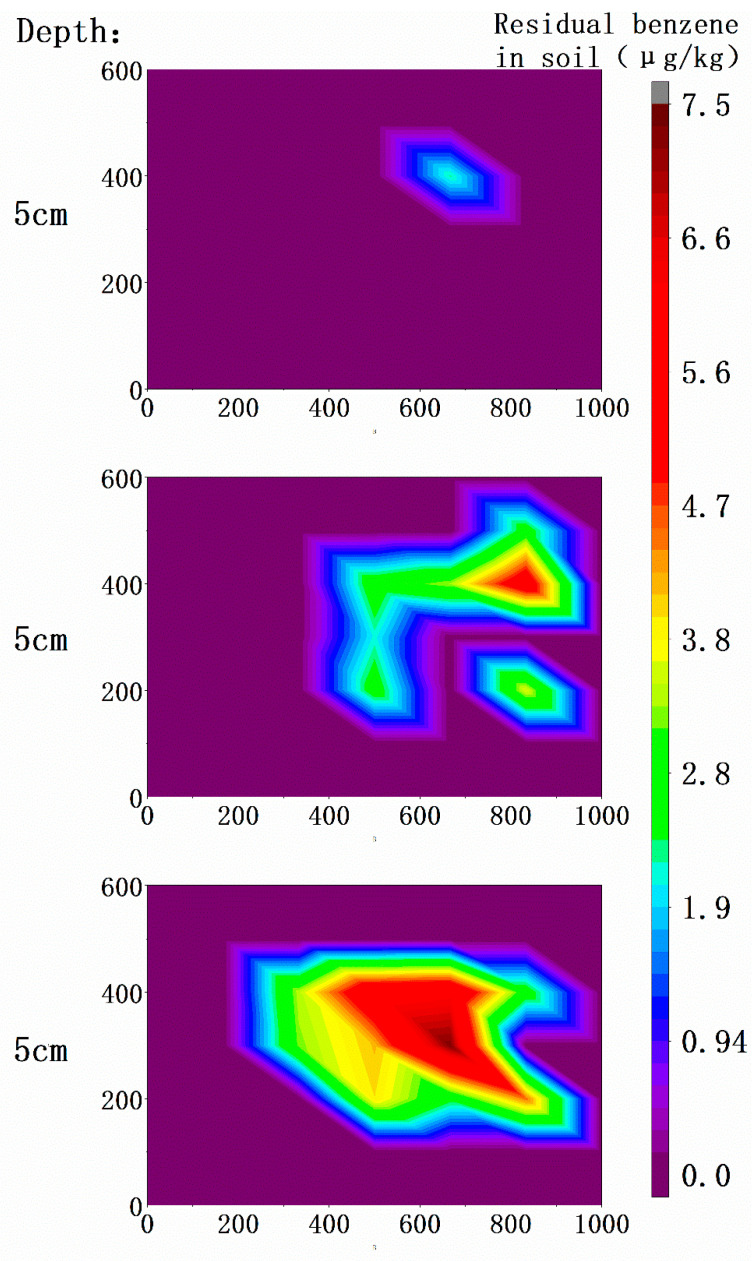
Distribution map of residual benzene in soil.

In this research, 75 treated soil samples were collected in three layers. The benzene content in soil was detected by gas chromatography. The detection results showed that most of the sites were not detected (the detection limit was 1.9 μg∙mg^−1^). The highest detection content was 7.3 μg∙kg^−1^, which was 0.04% of the initial soil concentration. Similarly, the contour map of benzene content in the treated soil was drawn with the left front end of the device as the origin. The result is shown in Figure 6. The distribution of benzene residues in treated soil was low in surface layer, high in bottom layer, high in center, and low around.

Soil has high specific heat capacity; it is a poor conductor of heat. In T-SVE, direct thermal contact heating with resistance is usually a slow process [24]. At the beginning, the soil was just stirred with water, the overall temperature was relatively uniform, about 28 °C. After the system was heated up for 90 min, the temperature of the middle soil layer around the heating rod began to change obviously. At 240 min, the temperature of most sensors reached the predetermined 80 °C. After 1200 min, the highest temperature in the middle soil layer reached 110 °C, then the temperature and its distribution basically remained in this state. On the plane, the temperature distribution was obviously higher around the heating rod and lower around it. It was worth noting that at about (200, 100) and its four symmetrical positions, although the position is closer to the heating rod, the temperature was lower most of the time, especially after the ventilation starts at 240 min. This is because the ventilation pipe is near there, the ventilation pipe has obvious influence on its heat conduction nearby. After the start of ventilation, cold air enters the soil continuously. This shows that the influence of ventilation on temperature is obvious. However, the temperature at the center extraction pipe did not drop significantly due to the start of extraction. This may be because extraction concentrates the energy of the system to the middle area through thermal convection. The box is provided with four heating rods with symmetrical centers. Therefore, on the plane, there are four obvious high temperature centers. In the vertical direction, because the heating rod is 5 cm deep and 25 cm long, the temperature field is obviously high in the middle soil layer and low in the top and bottom soil layers. The temperature of topsoil is slightly lower than that of subsoil because of contact with air above and condensed water on the tank cover.

After the soil was filled, the benzene concentration in the soil gas was uniform, 1000 mg∙m^−3^. At the beginning of the heating stage, the benzene concentration changed obviously, the overall concentration almost doubled every 20 min. At 90 min, the central concentration reached about 8500 mg∙m^−3^, and the corner concentration was also higher than 4000 mg∙m^−3^. At this moment, the distribution of benzene concentration in the middle soil layer was almost the same as that of temperature. As the diffusion of soil gas is obviously faster than the transfer of temperature in soil, although the temperature of surface and bottom soil is still low, the benzene concentration in soil gas is almost the same as that in the middle layer. At 180 min, the concentration reached its peak, the distribution of high concentration in the center and low concentration around it reached the extreme meanwhile. The highest concentration in the center reached 11,200 mg∙m^−3^, with an average value of 7521.3 mg∙m^−3^. At this time, the distribution was slightly lower in the middle and higher around on the plane. Considering the rapid diffusion of soil gas, it was speculated that the benzene around the heating rod was almost completely thermally desorbed at 180 min, but the benzene in the surrounding soil was still desorbing. At 240 min, the overall concentration decreased to about 7500 mg∙m^−3^, with an average value of 7629.4 mg∙m^−3^, slightly higher than that at 180 min. This indicated that the thermal desorption between these two times was inefficient or nearly completed, and the change of soil gas in this stage was mostly caused by the free diffusion of soil gas. Li [25] removed benzene from the soil through a similar stirable steam-enhanced SVE device, and the properties of the tested soil were similar to this study. The study found that at 80 °C, adding mineral powder and stirring, with a gas flow rate of 6 L∙min^−1^, the removal rate of benzene reached 90% within 2 h. This was basically consistent with the change of soil gas concentration in the early stage of heating in this treatment.

**Figure 7:**
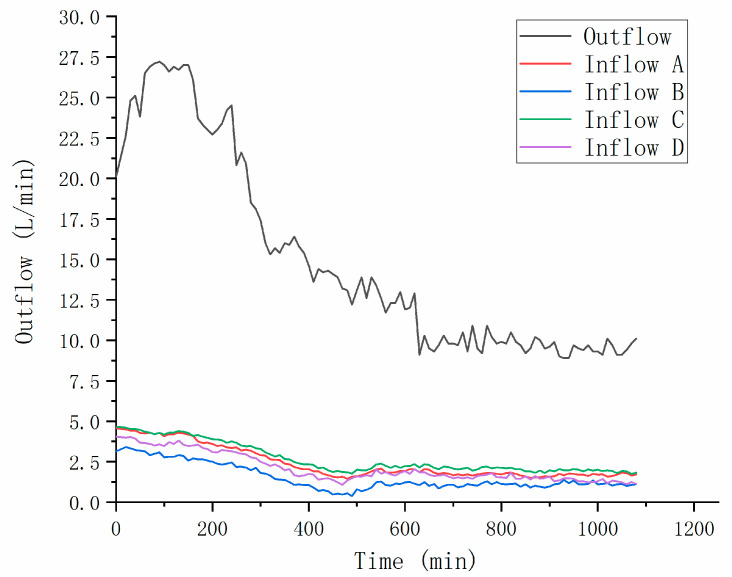
Inflow and outflow over time.

**Figure 8:**
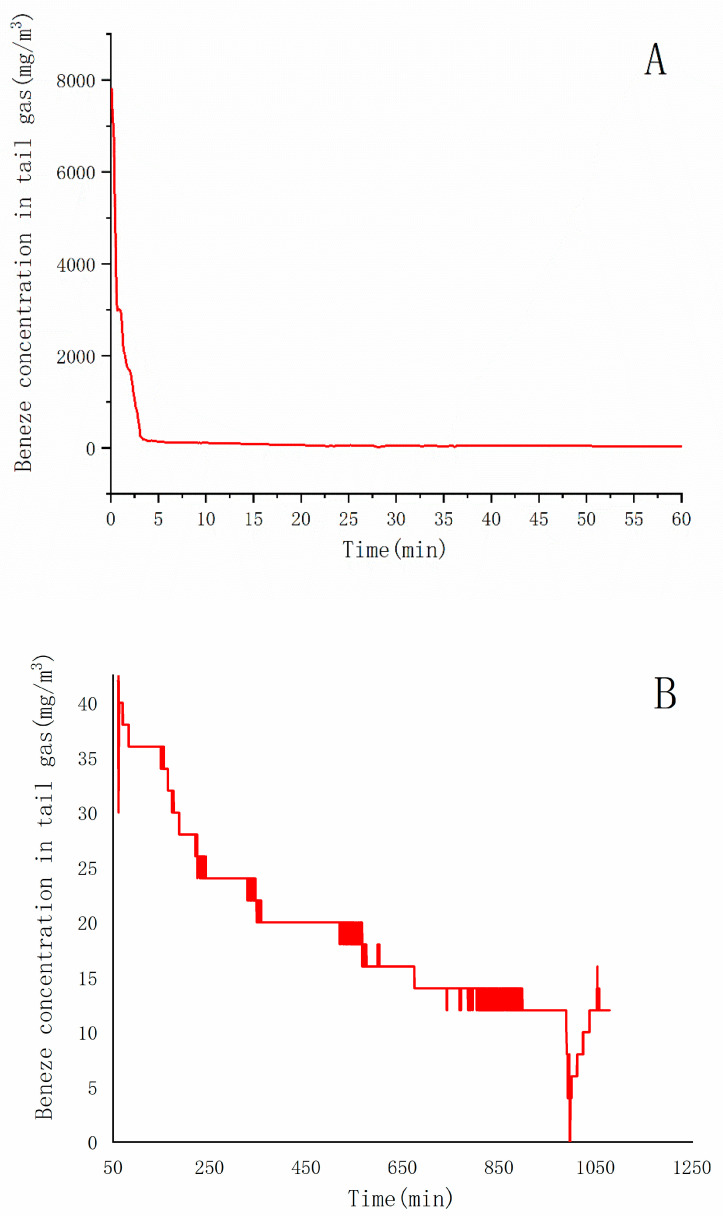
Changes of benzene concentration in tail gas over 60 min (**A**) and 60 to 1200 min (**B**).

After the extraction started, the benzene concentration in the system decreased rapidly in a very short time. As a result, in the contour map of 250, 260 and 400 min, the scale of color scale had to be changed in order to show the distribution change of concentration obviously. After extraction for 10 min (total process of 250 min), the concentration of all monitoring points was lower than 1000 mg∙m^−3^. After 20 min (260 min), the whole concentration decreased by two orders of magnitude, the distribution in plane space conformed to the law of central extraction, it had no obvious correlation with the distribution of temperature. It was worth noting that the concentration in the upper right corner of the device was abnormally high. Combined with the analysis of inlet flow in Figure 7, it was deduced that the ventilation condition of the upper right ventilation well was inferior to that of other ventilation wells. At 400 min, the system concentration was relatively uniform, all below 100 mg∙m^−3^, with the central concentration about 40 mg∙m^−3^ and the lowest concentration reaching 14 mg∙m^−3^, it was basically consistent with the monitoring situation of tail gas benzene concentration in Figure 8. In the extraction stage, there was no significant difference of benzene concentration in the vertical direction, and a slow decline in the upper middle layer and a rapid decline in the middle bottom layer. Liao [26] used a two-dimensional columnar experimental device to perform nonthermally enhanced SVE on benzene contaminated soil, and found that the removal rate was only 80% after 45 h at a gas flow rate of 15 L∙min^−1^. Moreover, because the vent holes were arranged on the outer side of the lower end, there was an obvious dominant channel, and the benzene content of the inner soil had obvious residues. In this study, the design of the extraction pipe was improved, and the position of the ventilation pipe was optimized to prevent the occurrence of dominant channels as much as possible. Thanks to it, the dominant channel effect was not obvious in the treatment.

Combined with the residual benzene concentration in the treated soil, further analysis was made. On the plane, there was basically no detection in the outermost periphery of the three soil layers, and the residual benzene concentration was higher in the middle and lower in the periphery, higher in the right side and lower in the left side. The reason for the imbalance between the left and right residues was the differences in the ventilation of the ventilation pipe. The lower ventilation flow obviously slowed down the extraction process and reduced the final treatment effect. Therefore, it was speculated that the critical extraction flow rate of benzene in the completely treated soil may be in the middle of C pipe and B pipe flow rate in this experiment. Although there was a big difference in benzene residues between topsoil and bottom soil, the difference in temperature was small. It also showed that in this experiment, the temperature should exceed the critical temperature for completely treating benzene in this soil, which was not a limiting factor of the remediation.

### 3.2. Airflow Changes and Remediation Efficiency

In the experiment, the running power of vacuum pump was adjusted to the maximum. It was found that the flowmeter at the tail end of pneumatic pipeline showed only 22.1 L∙min^−1^, this was significantly lower than that at idling. After confirming that the pipeline setting and airtightness were all right, it was speculated that the resistance of soil system in the box is large. In the follow-up monitoring, it was found that some obvious changes occurred in the flow rate, so the flow rate was monitored and recorded. The acquisition used the mobile phone time-lapse shooting function, it was set to shoot once every 10 min, recording the flow change every 10 min, with a total of 108 records.

The inflow numbers are a, b, c, and d, which correspond to the vents of upper left, upper right, lower left, and lower right ventilation wells, respectively. The changes in the flow rate of each in flow and the outflow rate are shown in Figure 7. It can be seen that the extraction flow rate began to increase significantly after the extraction began; It reached its peak of 28.9 L∙min^−1^ at about 140 min, and then began to decrease significantly. It reached a low point of 10.2 L∙min^−1^ at about 640 min, and then fluctuated up and down at this value until the end of the test.

No similar experiment has mentioned the change of air velocity during the treatment. Theoretically, extraction and heating take away water from the system, the soil porosity should be increased, the air resistance should be reduced, and the flow rate should be gradually increased. This situation of rising first and then falling is presumably because the vacuum pump continues to operate, this causes the equipment to overheat and reduces the operating efficiency of the electric motor. The sum of the flow rate of the inflow is lower than the pumping flow in the whole process, especially at the beginning of extraction. One of the possible reasons is that the negative pressure in the box after the start of extraction makes the front sampling hole or the port reserved at the top air leak. Among the vents, the flow rate of port B on the right is significantly lower than other vents in the whole process, and the aeration capacity of the surface B pipe is inferior to other vent pipes, which affects the distribution of soil gas benzene concentration and soil residual benzene distribution.

After heating for 4 h, the extraction started, and after the vacuum pump, a PID detector was connected in parallel to monitor the benzene in the tail gas. After testing, connecting the detector in parallel at this position can effectively monitor the tail gas concentration. Compared with the monitoring data of the detector directly connected to the extraction pipe outlet, the error is less than 1%. Due to the limitation of equipment storage, and for the convenience of calculation, the detector is set to record data once every 6 s to represent the average gas concentration in the first 6 s, and a total of 10,800 data were recorded.

The change of benzene concentration in tail gas during the test is shown in Figure 8. As the concentration changes greatly with time, the data is divided into two parts: the first hour (Figure 8A) and the last 19 h (Figure 8B). It can be seen from the figure that the benzene concentration in the tail gas at the beginning of extraction is extremely high, reaching 7812 mg∙m^−3^. Once extraction started, the concentration dropped sharply. It dropped to 100 mg∙m^−3^ after 11.7 min. After about 60 min, it decreased to 35 mg∙m^−3^ and maintained for about 200 min. After that, it gradually decreased, and finally remained at 15 mg∙m^−3^.

The method of heating first and then extracting leads to the extremely high benzene concentration in soil gas in the box at the beginning of extraction. This shows that the system has basically completed the thermal desorption of benzene and maintained the dynamic equilibrium at high temperature. After the extraction started, the air containing benzene was pumped out quickly, and completed 43% of the pollutant extraction volume in 2% of the total treatment process.

After 60 min, the benzene concentration in the extracted gas was still about 45 mg∙m^−3^. At this time, the extraction can still effectively remove the benzene concentration in the box. At 600 min, the benzene concentration in the exhaust gas reached 15 mg∙m^−3^ and basically no longer decreases. The amount of pollutants extracted from 60 to 600 min accounts for about 45% of the total.

According to the results of soil monitoring before the test, the total mass of soil benzene is about 5340. Based on the Formula 1 calculation, approximately 5389 mg of benzene was extracted at the end of the treatment and the removal rate was 100.9%.

The benzene concentration test results of the treated soil showed that benzene was detected at 18 points. The maximum content is 7.3 μg∙kg^−1^, which is 0.04% of the 17.8 mg∙kg^−1^ soil content before the treatment. After treatment, the average residual benzene concentration of the soil is only 0.916 μg∙kg^−1^, the total content is about 274.8 μg, and the removal rate reaches 99.995%

In a similar device experiment, Li [27] adopted a one-dimensional columnar device for thermally enhanced SVE of benzene-contaminated soil, which was first thermally enhanced and then extracted. The study showed that in 7 days after heat intensification at 80 °C and extraction at 3 L min^−1^ for 12 h, the removal rate of benzene with an organic content of 3.5% soil reached 93%. The organic matter content of the tested soil was 7.5%, which was not suitable for gas-phase extraction [28]. However, due to better heat preservation conditions and higher heating power, and the benzene removal rate was over 99% within 24 h.

It may bring some significance for further simulation research and engineering application. It means that for volatile organic compounds with a lower boiling point, heating throughout the process may be unnecessary and uneconomical. In addition, heating first and then pumping may be of great help in saving pumping costs and treatment time. In the follow-up research, more detailed energy and economic analysis, more types of pollutants, diversified pollution situations, and comparative study of computer models should be included, in order to obtain important parameters for the engineering application of heat conduction enhanced SVE and build a generalized model for site remediation.

## 4. Conclusions

In this study, the box device was designed and assembled, and the remediation of soil with benzene of 17.8 mg∙kg^−1^ was completed by single extraction pipe, four heating rods and four aeration pipes. The removal rate of benzene in this treatment was 99.995%. Under the heating of a total of 4000 W heating rods, the overall temperature of the system raised to 80 °C in 4 h, and the temperature distribution was uniform. The treatment completed 48% benzene extraction with 2% of the total time. The treatment took 10 h to reach the trailing stage.

In general, the device can realize an effective simulation of heat-conducting thermally enhanced SVE. Compared with experiments with similar devices, this treatment scheme can more efficiently remove benzene from the soil. The modular design of the device has good scalability, so relevant research could be sustainable.

## Figures and Tables

**Table 1:** Basic properties of the soil used.

Bulk Density (g/cm^3^)	Porosity (%)	Organic Matter Content (%)	Silicon Content (mg∙g^−1^)
1.74	35.8	7.5	344

## Data Availability

The data presented in this study are available on request from the corresponding author.

## References

[1] Tian F., Gao P., Tao J., Qi J., Zhu X. (2019). Research on Environmental Impact of Remediation of Industrial Contaminated Sites. J. Green Sci. Technol..

[2] Hentati O., Lachhab R., Ayadi M., Ksibi M. (2013). Toxicity assessment for petroleum contaminated soil using terrestrial invertebrates and plant bioassays. Envion. Monit. Assess..

[3] Khan M.A.I., Biswas B., Smith E., Naidu R., Megharaj M. (2018). Toxicity assessment of fresh and weathered petroleum hydrocarbons in contaminated soil—A review. Chemosphere.

[4] Ramadass K., Megharaj M., Venkateswarlu K., Naidu R. (2015). Ecological impli-cations of motor oil pollution: Earthworm survival and soil health. Soil Biol. Biochem..

[5] Tang J., Wang M., Wang F., Sun Q., Zhou Q. (2011). Eco-toxicity of petroleum hy-drocarbon contaminated soil. J. Environ. Sci..

[6] Simpanen S., Yu D., Mäkelä R., Talvenmäki T., Sinkkonen A., Silvennoinen H., Romantschuk M. (2018). Soil vapor extraction of wet gasoline-contaminated soil made possible by electroosmotic dewatering—Lab simulations applied at a field site. J. Soils Sediments.

[7] Ma J., Lahvis M. (2020). Rationale for Soil-Gas Sampling to Improve Vapor Intrusion Risk Assessment in China. Ground Water Monit. Remediat..

[8] Ma J., Jiang L., Lahvis M.A. (2018). Vapor Intrusion Management in China: Lessons Learned from the United States. Environ. Sci. Technol..

[9] USEPA (2007). Treatment Technologies for Site Cleanup: Annual Status Report.

[10] James J.B., David J.W. (1994). Soil Clean Up by In-Situ Aeration. XIV Effects of Random Permeability Variations on Soil Vapor Extraction Clean-Up Times. Sep. Sci. Technol..

[11] Yoon H., Kim J.H., Liljestrand H.M. (2002). Effect of water content on transient nonequilibrium NAPL—Gas mass transfer during soil vapor extraction. J. Contam. Hydrol..

[12] Wilson D.J., RodríGuez-Maroto J.M., Goamez-Lahoz C. (1994). Soil cleanup by in-situ aeration. XIX. Effects of soil age on soil vapor extraction remediation rates. Sep. Sci. Technol..

[13] Wilson D.J., Gómez-Lahoz. C., Rodríguez-Maroto J.M. (1994). Soil cleanup by in-situ aeration. XVI. Solution and diffusion in mass-transport-limited operation and calculation of darcy’s constants. Sep. Sci. Technol..

[14] Trvais C.C., Mcinnis J.M. (1992). Vapor extraction of organics from subsurface soils. Environ. Sci. Technol..

[15] Poppendieck D.G., Loehr R.C., Webster M.T. (1999). Predicting hydrocarbon removal from thermally enhanced soil vapor extraction systems 1, Laboratory studies. J. Hazard. Mater..

[16] Lim M.W., Lau E.V., Poh P.E. (2016). A comprehensive guide of remediation technologies for oil contaminated soil-present works and future directions. Mar. Pollut. Bull..

[17] Poppendieck D.G., Loehr R.C., Webster M.T. (1999). Predicting hydrocarbon removal from thermally enhanced soil vapor extraction systems 2. Field Study. J. Hazard. Mater..

[18] Rathfelder K., Lang J.R., Abriola L.M. (1995). Soil vapor extraction and bioventing: Applications, limitations, and future research directions. Rev. Geophys..

[19] Roland U., Bergmann S., Holzer F., Kopinke F.-D. (2010). Influence of in situ steam formation by radio frequency heating on hemadsorption of hydrocarbons from contaminated soil. Environ. Sci. Technol..

[20] Reising L., McCartney J.S. (2016). Laboratory Evaluation of Low-Temperature Thermally-Enhanced Soil Vapor Extraction. Geo-Chicago.

[21] Shao Z. (2015). Pollutant Removal during the Thermally Enhanced Soil Vaper Extraction.

[22] Tasca A.L., Clematis D., Panizza M., Vitolo S., Puccini M. (2020). Chlorpyrifos removal: Nb/boron-doped diamond anode coupled with solid polymer electrolyte and ultrasound irradiation. J. Environ. Health Sci. Eng..

[23] Li X. (2018). Process Design and Research on Operating Parameters of Thermally Enhanced Sve Method to Repair Hydrocarbon Contaminated Soil.

[24] Yu Y., Liu L., Yang C., Kang W., Yan Z., Zhu Y., Wang J., Zhang H. (2019). Removal kinetics of petroleum hydrocarbons from low-permeable soil by sand mixing and thermal enhancement of soil vapor extraction. Chemosphere.

[25] Li B., Zhu J., Ji M., Tu B., Lin X. (2016). Research upon Steam Enhanced Vapor Extraction for the Remediation of Benzene Homologues Contaminated Clayed Soil. J. Shanghai Jiaotong Univ. Agric. Sci..

[26] Liao Z. (2013). The Research about Remediation of Volatile Organic Contaminant by Thermal Enhanced Soil Vapor Extraction.

[27] Li P., Liao X., Yan X., Cui X., Ma D. (2014). Effect of Thermal Enhanced Soil Vapor Extraction on Benzene Removal in Different Soil Textures. Environ. Sci..

[28] Lu Z., Pei Z., Lu S., Wang Y., Hu J., Yan S. (2011). Tetrachloride from soils by soil vapor extraction. Environ. Sci. Technol..

